# Expectations, Fears and Perceptions of doctors during Covid-19 Pandemic

**DOI:** 10.12669/pjms.36.COVID19-S4.2643

**Published:** 2020-05

**Authors:** Uzma Urooj, Asma Ansari, Asifa Siraj, Sumaira Khan, Humaira Tariq

**Affiliations:** 1 Uzma Urooj, MBBS, FCPS. Assistant Professor, Department of Gynae/Obs, NUMS, Army Medical College, MH Rawalpindi, Pakistan; 2 Asma Ansari, MBBS, FCPS. Associate Professor, Department of Gynae/Obs, Army Medical College, NUMS, CMH Rawalpindi, Pakistan; 3 Asifa Siraj Associate Professor, Department of Gynae/Obs, Army Medical College, MH Rawalpindi, Pakistan; 4 Sumaira Khan Assistant Professor, Department of Gynae/Obs, Army Medical College, MH Rawalpindi, Pakistan; 5 Humaira Tariq Assistant Professor, Department of Gynae/Obs, Army Medical College, CMH Murree, Pakistan

**Keywords:** Covid-19, Fears, Mental stress, Pandemic, Expectations

## Abstract

**Objectives:**

The aim of this study was to explore the expectations and fears faced by doctors during Covid-19 Pandemic.

**Methods:**

This is a mixed method exploratory survey. A questionnaire exploring expectations of doctors from administration and seniors as well as their fears while working during pandemic, was developed on Google survey Forms. It included eight closed ended questions and four open ended questions. Data was collected through online Google survey Forms during month of March and April 2020. Doctors were approached through email and WhatsApp group.

**Results:**

The mean age of participants was 33.58±4.21 years. Female 150(67.5%) and Male 72(32.4%) participated. 29(13.1%) Associate Professor, 34(15.3%) Assistant Professor, 56(25.2%) Senior Residents and 103(46.3%) residents, medical officers and house officers responded to the survey. 134(60.3%) doctors were working in hospitals which were not dealing with Covid-19. Fear included, infecting family members 177(79.7%), rapid spread of disease 140(63%), complications of disease 134(60.3%), becoming a carrier in 64(28.8%) and 62(27.9%) feared missing the diagnosis. More than 80% expected from seniors and administration, of providing PPE, facilitation, continue chain of supply of essential items, ensuring doctor safety, avoiding exposure of all doctors and keeping reserve workforce, limiting routine checkups, avoid panic and 20% had no expectations.

**Conclusion:**

It was concluded that doctors had their fears and perceptions regarding pandemic which need to be addressed while policy making. They fear wellbeing of their families and contacting Covid-19, if not provided proper PPE. Our study provides insight of expectations, fears and perceptions of our frontline which invariably gives insight of the views of healthcare workers.

## INTRODUCTION

End of 2019 brought the global community to be aware of an emerging health crisis which originated in WUHAN CHINA. Most of the world was not prepared for a situation in which their whole lives will change so in spite of warning messages, the response by global health community and policy makers was slow. In January 2020 the World Health Organization (WHO) declared the outbreak of a new coronavirus disease, COVID-19, to be a Public Health Emergency of International Concern.^1^ WHO stated that there is a high risk of COVID-19 spreading to other countries around the world. In March 2020, WHO made the assessment that COVID-19 can be characterized as a pandemic. So, after an initial mundane response suddenly a mass hysteria and panic were created. There was unfiltered non-scientific information bombardment^2^ which affected the health care professionals who are directly exposed to a new virus creating havoc. Reason being that they are not aware what they are fighting with no foreseeable cure and large number of their peers being affected or dying. Some health workers were facing stigmatization and even their families wanted to be disassociated due to a constant stream of negative news.^3^ Only recently the media has started giving statistics of recovered people too otherwise the news was filled with just people dying everywhere^4^ invariably contributing to stress and restlessness amongst doctors.

This outbreak is a unique and unprecedented scenario for most of the health workers and their families too. This is a long-term fight and requires sustained response.^5^ Staff needs to be protected from chronic stress and poor mental health during this response. This means they will have a better capacity to fulfil their roles. They need a regular up-to-date information and communication in order to avoid confusion and further stresses. The administrators are also facing the same stresses so there should be mutual trust, constant reassurance, availability of PPE, standard guidelines and flexible duty hours.^6^ Doctors are amongst the people most at risk of getting the disease. It is causing undue stress and restlessness when colleagues are sick or on ventilator in ICU due to coming in contact with Covid-19.^7^ Most of the doctors have no one else to take care of their children or families during times of self-isolation or quarantine. As every time a doctor falls ill the already strained health system gets a blow.

The biggest concern is bringing the virus home to their families. There is also a fear that if they fall ill they will be betraying the health system and their patients as they will not be able to contribute.^8^ As new information about the disease is upgraded daily the concerns also rise. The doctor realizes that being young will not be protective or the virus is airborne so from today the protection requirement is different.^9^ These challenging times are as hard for the patients, communities as well as the health care staff. There is a need to assess the fears, expectations and perceptions of health care workers in our context as it is a topic which is hardly given any attention. Doctors are there to treat but their views should be considered and addressed. This study was conducted to assess the perceptions, expectations and fears of doctors during the Covid-19 pandemic and identify the areas which need to be addressed.

## METHODS

A cross-sectional web-based survey was carried out by using google forms. A new questionnaire was developed, after validation from five experts, who were working as medical educationist as well as senior consultants with experience of more than 15 years. it was pilot tested. The final survey consisted of total 12 questions; 8 of which were close-ended and 4 were open-ended. Participants included the Pakistani as well as few UK 22.2% (n=10), USA 22.2% (n=10) clinicians, who were actively involved in clinical practice and duties during Covid-19 pandemic. Sample size was calculated by using open Epi calculator. These clinicians included anesthetist, surgeons, physicians, radiologists, pathologists, pediatricians, gynecologists, postgraduate trainees, medical officers & house officers working in various healthcare institutes. The diversity of the group allowed for generalization of results to all specialties to assess the clinicians. Opinions from all the angles. After ethical approval ER-A/28, and informed consent, 250 participants, selected through purposive sampling, were contacted through E-mails and WhatsApp group’s anonymity and confidentiality was assured. Later, reminders were also sent via e-mails and WhatsApp. 222 participants filled questionnaire and returned forms. Data was saved in Excel sheets directly from the Google forms. For close-ended questions frequencies and percentages were calculated. For open-ended questions inductive coding was done in excel. After initial coding, line-by-line coding was done for each response by primary researcher (UU) and the similar codes were grouped together to make themes. Frequencies were calculated for each theme. The coding process and themes were cross-checked by four reviewers separately (AA, AS, SK, RI) for validation.

## RESULTS

The study included 150(67.5%) Female and 72(32.4%) Male participants. The mean age was 33.58±4.21 years. 29(13.1%) Associate Professor, 34(15.3%) Assistant Professor, 56(25.2%) Senior Residents and 103(46.3%) residents, medical officers and house officers responded to the survey.

Covid-19 patients were not present in hospitals of 134(60.3%) doctors and 88(39.3%) doctors had Covid-19 patients in their hospitals. Fears of doctors are shown in Table-I.

**Table-I:** Fears of doctors.

Fear	Frequency
Infecting family members	177(79.7%)
Rapid spread of disease	140(63%)
Complications of disease	134(60.3%)
Becoming a carrier	64(28.8%)
Missing the diagnosis	62(27.9%)

Covid-19 patients were not present in hospitals of 60.36% (n=134) doctors who responded and 39.3% (n=88) doctors had Covid-19 patients in their hospitals. 83.3% (n=185) doctors were aware of the SOPs and policies related to Covid-19, whereas 15.8% (n=35) were not aware and 0.9% (n=2) responded “may be”. Donning and doffing awareness was there in 76.1% (n=169), no awareness in 5.8% (n=13) and “may be” was answer of 18.1% (n=40). Following themes were generated from the open-ended questions,

### Fears of doctor

Fears of doctors included, infecting family members 177(79.7%), rapid spread of disease 140(63%), complications of disease 134(60.3%), becoming a carrier in 64(28.8%) and 62(27.9%) feared missing the diagnosis. Some of the statements of participants were;

-“Facing a situation where I am helpless against the state of the patient” (P 2)
-“To get infected” (P 13)
-“We are working without PPE….may be infected with corona virus” (P 23)
-“To have the severe complications of the disease in the patients n in ourselves” (P 30)
-“…..Disease can spread rapidly” (P 35)
-“Fearful for the family back home” (P 40)
-“Treating a patient normally who turns out to be covid19 positive later on” (P 41)
-“I have to perform my duty because I am doctor…**no fear…”** (P 44)
-“I have faith in **Allah**” (P 45)
-“Exposing my family to infection as well” (P 49)
-“Catching the infection and unknowingly becoming a spreader rather than the care giver.” (P 178)
-“Asymptomatic carriers and transmission are worrisome” (P 180)
-“Panic among the paramedics and junior staff” (P 185)
-“As we work without PPE……fear of getting corona virus” (P=196)

### Feelings of doctors during duties

Doctors expressed different feelings while working during Covid-19 pandemic. Some of these feelings were; Uncertainty and fear 12.1% (n=27), sense of duty 27.9% (n=62), depressing circumstances 58% (n=129), anxiety 86% (n=191), worried 28.8% (n=64), motivated 33.3% (n=74), hopeful 56.7% (n=126), cautious 31% (n=69), ambitious 15.3% (n=34), apprehensive and confused 4% (n=9). Some of the statements were;

-“We feel proud of being the frontline fighters against this illness” (P 136)
-“As a doctor, I’m at high risk and I’m feeling proud while performing high risk duties during this pandemic” (P 153)
-“Praying to be out of this pandemic as early as possible” (P 169)
-“Feel grateful to be given a chance to help the affected” (P 176)
-“Fearful but ambitious” (P 181)
-“Uncertainty about the entire situation and fears of contacting infection” (P 188)
-“Worried because of not having proper PPE”
-“Feelings of empathy for people who are quarantined and for patients who have contracted covid-19” (P 193)
-“We have to work through thick and thin and protect our families as well” (P 197)
-“Working with full energy along with faith in Allah Almighty” (P 201)
-“Serving humanity and mankind” (P213)
-“I feel obliged that I am fulfilling my duties…” (P 220)
-“Situation is same everywhere. Protective kits, PPE is available to only the administration who will never directly see the patient, the real healthcare teams are given scanty sources”

**Fig.1:**
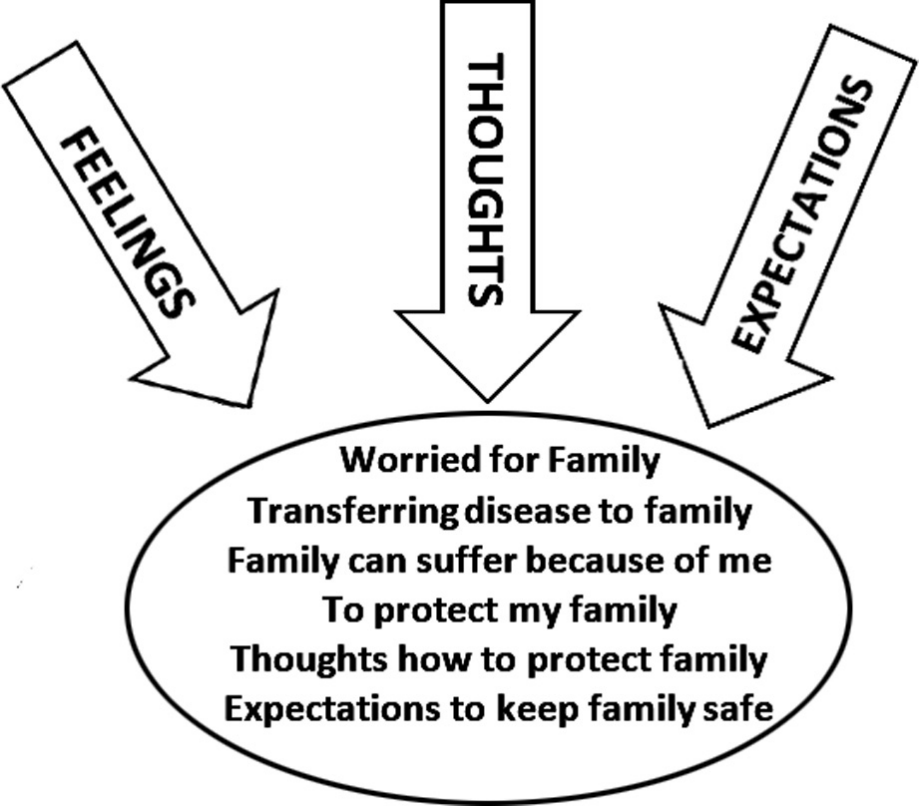
Family as center of concern.

### Thoughts going through the mind of doctors on way to duty

Responses of doctors on question regarding their thoughts while they are on way to hospital were mixed. Some of these thoughts are documented in their words as follows;

-“May ALLAH protect us all and give speedy recovery to the ill, and will I be getting proper PPE today or not” (P 07)
-“To adhere to SOP regarding patient handling” (P 11)
-“To be safe, responsible and stick to guidelines but there’s a hidden fear as well” (P 19
-“That I will get the disease somehow” (P 16)
-“I am a health care professional and it’s my duty to help patients suffering from diseases, and at the same time keep myself protected” (P 27)
-“Can this be avoided….is my duty warranted…or is it causing unnecessary exposure” (P 33)
-“Considering duties as our main responsibility being a doctor and having trust on Allah that He will protect us …” (P 41)
-“Nothing specific. Not always thinking about corona. Thinking about my juniors to keep their moral up” (45)
-“This time shall pass” (P 46)
-“Thinking of dealing a patient with covid19 positive but not knowing” (P 51)
-“That I don’t get to manage a critically ill Covid-19 patient” (P 58)
-“Thinking about its spread and protection by social distances” (P 63)
-“Here I come again Corona. You can’t stop me at home” (P 70)
-“About crisis will have to face” (P 73)
-“Another tough day dealing with the fear of covid-19, that I might not get infected and become a source for my family” (P 79)
-“My thoughts are towards performing my duties with full concentration and no negligence” (P 84)
-“Thinking about my mix feelings of fear and responsibilities” (P 103)
-“To work efficiently” (P 289)

### Expectations from administration, seniors and peers

There were many expectations from administration, seniors and peers. These are shown in Table-II.

**Table-II:** Expectations of doctors.

Expectations	Administration	Seniors	Peers
1.	To provide with PPE	Seniors to provide with clear guidelines	Colleagues to behave rationally and responsible
2.	Support for all staff	Role model	Empathetic
3.	Facilitation	Better SOPs and to stick to these SOPs	Not to panic
4.	Provide PPE to Doctors staff and max measures for the medical health professional’s life …	Duty hours should be minimized to have minimum exposure…	Support
5.	Don’t panic	Firm and humble	Cooperative
6.	Support, safe environment and provision of PPE	Role modelling and encouragement	Team spirit and emotional support
7.	Act sanely, exposing less staff at a time and have some staff in reserve for backup	Positive environment	Team work
8.	Limit normal routine checkups	Stand by us at all times, educate us on what to do, do all possible for the protection of their subordinates and patients	Keep each other motivated and help in terms of providing knowledge about corona and measure to protect each other

To quote few statements in which participants had no expectations from administration 39% (n= 86), such as;

-“I don’t have any expectations as this is a brand-new front, unprecedented, no one alive has fronted an issue of this magnitude….so I don’t expect policies to be perfect….and I know everyone has fear in their hearts…. naturally” (P 7)
-“No expectations from admin Our seniors and colleagues very cooperative we all will beat this war” (P 17)
-“Not a lot” (P 37)

More than 80% expected full support, provision of protective equipment, cooperation and organization from administration.

### Family

It won’t be wrong to say that 99% of doctors had feelings, thoughts and expectations centered towards their families.

## DISCUSSION

This survey enrolled 222 participants and gathered the data of doctors working in critical and uncertain conditions of pandemic. In this study significant number of participants expressed feelings of concern, anxiety, uncertainty and stress similar to other studies done which showed same psychological responses while working in high-risk situations.^10^ In our study age ranged from 26-53 years, 32.4% male and 67.5% females. In another study by Kang L et al 994 participants, including 183 (18.4%) doctors and 811 (81.6%) nurses, completed the survey. A total of 31.1% worked in high-risk departments. Majority of the participants were female (85.5%) similar to our study and age ranged 25 to 40 years (63.4%) which is also in agreement to our study. Whenever an epidemic hit there is an element of uncertainty and fear even amongst the health care providers which was evident in. This study as 36.3% of the participants had psychological fears.^11^

The fact that COVID-19 is human-to-human transmissible, associated with high morbidity, and potentially fatal may intensify the perception of personal danger.^12^ Same was the case in our study which showed that doctors while on their way to work thought of their personal protection, avoiding being a carrier, keeping spirits up and sense of responsibility. In our study fear of complications of disease in patients was 60.36%, 79.7% feared infecting family members, 27.9% not diagnosing Covid-19 positive patient, 63% rapid spread of disease and 28.8% becoming a carrier. A survey conducted in China during the initial outbreak of COVID-19 found that 53.8% of respondents rated the psychological impact of the outbreak as moderate or severe, 16.5% reported moderate to severe depressive symptoms, 28.8% reported moderate to severe anxiety symptoms, and 8.1% reported moderate to severe stress levels.^13^

Health Care Workers working in emergency departments, intensive care units, and isolation wards had a greater risk of developing adverse psychological outcomes than those of other departments, because they were directly exposed to the infected patients in a highly demanding environment.^14^

Expectations of most of the doctors from administration were to provide full support, protective equipment provision, planning and logical distribution of resources in order to Gare up for future uncertainty. First and foremost, organizational leaders should provide clear messages that clinicians are valued and that managing the pandemic together is the goal.^15^

Although global health crises share common characteristics across national contexts, each country has its unique political and social systems that affect information behaviors and environments.^16^ Our health care workers expected seniors and peers to be more empathetic, cooperative, not to panic, show team work, role modelling and support. Few studies have showed that challenges included the need to frequently adjust build to meet rapidly evolving requirements, communication and adoption, and coordinating the needs of multiple stakeholders while maintaining high-quality medical care.^17^ Support from healthcare authorities, regulators, and the government for doctors making difficult clinical decisions is vital, as is the understanding that they will be supported in the event of adverse outcomes.^18^ Perceptions of doctors in this study were Uncertainty and fear 12.1%, sense of duty 27.9%, depressing circumstances 58%, anxiety 86%, and worried 28.8%. Such stressful conditions can lead to substantial depression in short term and a much more risk of burn out in long term. Another cross-sectional, survey-based study collected demographic data and mental health measurements from 1257 health care workers in 34 hospitals from January 29, 2020, to February 3, 2020, in China. A total of 1257 of 1830 contacted individuals completed the survey, with a participation rate of 68.7%. A total of (64.7%) were aged 26 to 40 years, and (76.7%) were women. Of all participant (60.8%) were nurses, and (39.2%) were physicians. (60.5%) worked in hospitals in Wuhan, and 522 (41.5%) were frontline health care workers. A considerable proportion of participants reported symptoms of depression (50.4%), anxiety (44.6%), insomnia (34.0%), and distress (71.5%).^7^

All doctors showed concern for carrying the disease to their families and expected support from seniors and administration, leaders should aim to monitor clinician wellness and proactively address concerns related to the safety of clinicians and their families.^19^ COVID-19 rapidly spread from a single city to the entire country in just 30 days in China. The sheer speed of both the geographical expansion and the sudden increase in numbers of cases surprised and quickly overwhelmed health services all over the world.^20^ Need of time is to consider as well as plan before hand and to consider views of our doctors as pertinent.

### Limitations and Clinical Implications

This study was conducted during pandemic and lockdown. There is a need to carry out more studies to explore the environment and working conditions of healthcare workers. It will help policy makers and administration to take appropriate decisions and work as team with motivation during this crisis.

## CONCLUSION

Covid-19 pandemic has raised numerous challenges all around the world. The health care system is at breaking point in many developed countries. Keeping opinion of doctors in view we need to plan as quickly as possible, to identify how we can reconfigure our services. Our frontline medical staff need to be protected both mentally and physically, which can be achieved by working together as a team.

### Authors’ Contribution

**UU:** Conceived, designed, data analysis and editing of manuscript

**AA:** Data analysis and editing of manuscript

**AS and SK:** Data collection/analysis.

**HT:** Data collection.

**UU:** Takes the responsibility and is accountable for all aspects of the work in ensuring that questions related to the accuracy or integrity of any part of the work are appropriately investigated and resolved.
